# Comparison between the efficacies of curcumin and puerarin in C57BL/6 mice with steatohepatitis induced by a methionine- and choline-deficient diet

**DOI:** 10.3892/etm.2013.1461

**Published:** 2013-12-24

**Authors:** YUNLIANG WANG, JIAN LI, LI ZHUGE, DONGMEI SU, MEIJUAN YANG, SHIYING TAO, JUNXIANG LI

**Affiliations:** 1 Dongfang Hospital, Beijing University of Chinese Medicine, Beijing 100078, P.R. China; 2 Preclinical College, Beijing University of Chinese Medicine, Beijing 100029, P.R. China

**Keywords:** puerarin, curcumin, steatohepatitis, peroxisome proliferator-activated receptor-γ, nuclear factor-κB

## Abstract

Non-alcoholic fatty liver disease (NAFLD) is a prevalent disease, which features an abnormal accumulation of lipids inside hepatocytes. Steatohepatitis plays a critical role in the process resulting in liver fibrosis and cirrhosis. Curcumin and puerarin are herbal products widely used in Asia, which are believed to have therapeutic benefits for alleviating the symptoms of steatohepatitis. In this study, mice models of steatohepatitis induced by a methionine- and choline-deficient diet (MCD) were established to compare the pharmacological actions of curcumin and puerarin. The results showed that curcumin and puerarin exerted inhibitory effects against MCD-induced steatohepatitis in mice. Briefly, curcumin and puerarin significantly downregulated the levels of tumor necrosis factor-α in the blood serum of mice (P<0.01, versus the MCD group). In addition, the levels of triglycerides, total cholesterol and low density lipoproteins in the serum were significantly reduced by puerarin treatment (P<0.05, versus the MCD group). The concentration of interleukin-6 was downregulated by curcumin only (P<0.01, versus the MCD group). Curcumin and puerarin significantly increased the levels of peroxisome proliferator-activated receptor-γ (PPARγ; P<0.05, versus the MCD group). Moreover, increased nuclear factor-κB (NF-κB) was markedly attenuated by curcumin (P<0.05, versus the MCD group). In conclusion, curcumin and puerarin appear to exert different actions against steatohepatitis. It is possible that puerarin regulated lipid metabolism in the ‘first hit’ stage through the PPARγ pathway, while curcumin inhibited the inflammatory response in the ‘second hit’ stage through the NF-κB pathway.

## Introduction

Non-alcoholic fatty liver disease (NAFLD), commonly known as fatty liver, is a disease characterized by an abnormal accumulation of fats [triglycerides (TGs)] inside liver cells (1). Thus far, the prevalence of NAFLD has consistently increased with lifestyle changes (2). Steatohepatitis is histologically characterized by a significant accumulation of hepatic lipids and lobular necro-inflammation in NAFLD, which may be progressive and eventually induce liver fibrosis and cirrhosis (3,4).

Although much progress has been made in recent years, the pathogenesis of steatohepatitis has not been fully elucidated. It is known that defects in fat metabolism are responsible for the pathogenesis of NAFLD, which may be due to an imbalance in lipid storage and consumption in hepatocytes. Peroxisome proliferator-activated receptor-γ (PPARγ) signaling is important in hepatic fat accumulation, transport and utilization (5). PPARγ, also known as the thiazolidines ligand, plays a critical role in regulating fatty acid storage and metabolism. A previous study identified that PPARγ exhibited an anti-steatohepatitic effect by reducing the production of hepatic pro-inflammatory cytokines, such as tumor necrosis factor-α (TNF-α) and interleukin-6 (IL-6) (6). Furthermore, the activation of PPARγ may inhibit inflammatory responses by preventing the activation of nuclear transcription factors, such as nuclear factor-κB (NF-κB) (7). For these reasons, PPARγ may be a potential therapeutic target in the treatment of steatohepatitis. Additional clinical studies have demonstrated that PPARγ agonists, such as thiazolidinediones (TZDs), not only increase insulin sensitivity in adipose, liver and skeletal muscle tissue, but also affect hepatic fat accumulation by enhancing fatty acid oxidation (8,9). Despite their validated efficacy and widespread use, TZDs possess a number of side-effects, including significant weight gain and peripheral edema (10,11). In serious cases, TZDs may result in severe hepatotoxicity (12). In this regard, it is necessary to develop novel agents that target PPARγ, but with reduced adverse effects.

**Figure 1:**
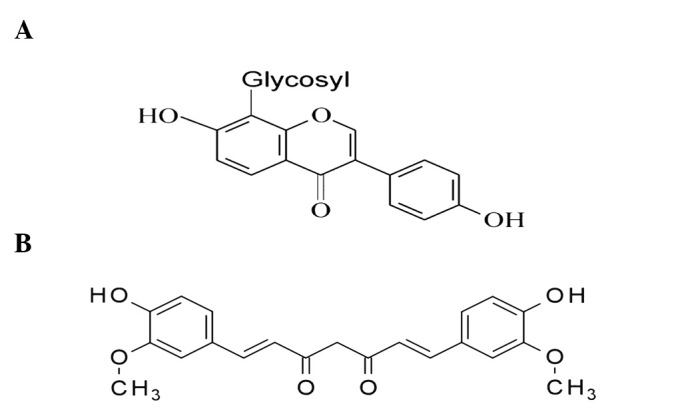
Chemical structures of puerarin (A) and curcumin (B).

Medicinal plants, also called herbal medicines, have been traditionally used for treating liver disease worldwide (13). There are numerous herbal products that are believed to have therapeutic benefit on NAFLD, such as silymarin (milk thistle), glycyrrhizin (licorice root extract) and curcumin (turmeric extract) (14). Among them, curcumin (Fig. 1B) is the best known and has been used for centuries in Asia as a dietary spice, a food coloring and a treatment for inflammation, wounds, and gastrointestinal and liver disorders (15). A previous study demonstrated that curcumin may alleviate steatohepatitis and inhibit liver fibrogenesis (16). The possible mechanisms of curcumin may involve the stimulation of PPARγ activity and the inhibition of hepatic stellate cell (HSC) activation (17).

Previous studies have investigated the anti-oxidative and anti-inflammatory activities effects of puerarin (Fig. 1A) (18). Puerarin has been shown to exhibit therapeutic effects on NAFLD by acting as an antioxidant, lowering cholesterol levels and improving leptin signal transduction (19,20). In the present study, the intervening actions of puerarin and curcumin were compared on mice models of steatohepatitis induced by a methionine- and choline-deficient (MCD) diet. With regard to previous studies concerning the roles of curcumin and puerarin in PPARγ signaling, it may be hypothesized that curcumin and puerarin have potential as therapeutic agents for the treatment of steatohepatitis.

## Materials and methods

### Chemicals and reagents

Feeds for the methionine-choline-sufficient (MCS) diet and methionine-choline-deficient (MCD) diet were provided by the Trophic Animal Feed High-tech Co., Ltd. (Nantong, China). Curcumin and puerarin were purchased from Xi’an Guanyu Bio-tech Co., Ltd. (Shaanxi, China). Colorimetric kits for the testing of triglyceride (TG), total cholesterol (TC), high density lipoprotein (HDL) and low density lipoprotein (LDL) were purchased from BioSino Bio-technology and Science, Inc. (Beijing, China). Enzyme-linked immunosorbent assay (ELISA) kits for TNF-α and IL-6 and were purchased from Shanghai Yanji Biotechnology Co., Ltd. (Shanghai, China). Anti-NF-κB (p65, ab31481), anti-PPARγ (ab27649), and anti-GAPDH antibodies (ab9483) were purchased from Abcam (Cambridge, UK). Additional reagents were obtained from Sigma-Aldrich (St. Louis, MO, USA).

### Animal handling procedure

Thirty-two C57BL/6 mice (weight, 20–25 g) were purchased from Vital River Laboratories (Beijing, China; certificate no. SCXK-2006–0009) and divided into four groups: The normal control group, the model control group, the curcumin treatment group and the puerarin treatment group. Mice were housed in a temperature-, humidity- and light-controlled environment (temperature, 22±2°C, a 12-h light/dark cycle, 50–60% humidity) with access to rodent feed and water *ad libitum*. Normal control mice were fed the MCS diet and the remaining mice were fed the MCD diet for 2 weeks.

### Drug administration

Curcumin and puerarin were administered orally in a volume of 0.1 ml/10 g body weight once a day for 2 weeks at the same time as MCD feeding. Drugs were dissolved in dimethyl sulfoxide (DMSO) and then diluted in distilled water to a concentration of 90 mg/ml (18,21). Vehicle solution (DMSO mixed with distilled water) was administered to the normal control mice. The experimental procedures were reviewed and approved by the Animal Care and Use Committee in the Beijing University of Chinese Medicine (Beijing, China) prior to the animal experiments being performed.

### Detection of TG, TC, HDL and LDL levels in the blood serum

Blood was harvested after the mice were anesthetized. At the end of treatment, animals were anesthetized using 4% chloral hydrate after a 12-h over night fast. The blood samples were obtained from the inferior vena cava. Following centrifugation at 644 × g for 10 min at 4°C, the serum was collected to measure the levels of TG, TC, HDL and LDL. All these measurements were determined using enzymatic colorimetric kits and were performed according to the manufacturer’s instructions.

### Histopathological analysis

One fresh section of liver tissue from each mouse was kept in liquid nitrogen for 4–10 sec for the running frozen section technique. Sections were stained with Oil red O (Sigma-Aldrich). An additional section of liver tissue was fixed by immersion in 10%-buffered formalin for paraffin embedding, and hematoxylin and eosin staining.

### ELISA for the detection of TNF-α, and IL-6 levels in the serum

The concentrations of TNF-α and IL-6 in the serum of the mice were analyzed using commercially available ELISA kits according to the manufacturer’s instructions. Briefly, all the samples were diluted at 1:10. The absorbance was read at 450 nm using a microplate reader (Multiskan MK3; Thermo Scientific, Rockford, IL, USA). Samples and standards were run three times.

### Western blotting to detect PPARγ and NF-κB expression

Proteins in the liver tissue homogenates were extracted using ice-cold tissue lysis buffer. Protein concentrations were determined using a BCA protein assay kit (Promega, Madison, WI, USA). Samples were separated by 10% SDS-PAGE and transferred onto polyvinylidene difluoride membranes. The membranes were immunoblotted with primary antibodies that recognized PPARγ (1:2,000), NF-κB (1:400) and GAPDH (1:5,000). Peroxidase-conjugated secondary antibodies [goat polyclonal secondary antibody to mouse (ab6006); Abcam] and an enhanced chemiluminescence detection system [ECL Western Blotting Substrate Kit - 500 Tests (ab65628); Abcam] were used according to routine methods. The intensities of the protein bands were analyzed using Gel-Pro Analyzer, version 3.2 software (Bio-Rad Gel Doc 2000 digital gel imaging system; Bio-Rad, Hercules, CA, USA). GAPDH protein was used as the internal control to normalize for protein loading.

### Statistical analysis

Data are expressed as the mean ± standard error. Differences between the mean values of normally distributed data were assessed using a one-way analysis of variance and the Student-Newman-Keuls test. Analyses were performed using Excel and Paint software for Windows. P<0.05 was considered to indicate a statistically significant difference.

## Results

### Effects of puerarin and curcumin on lipid metabolism

**Figure 2:**
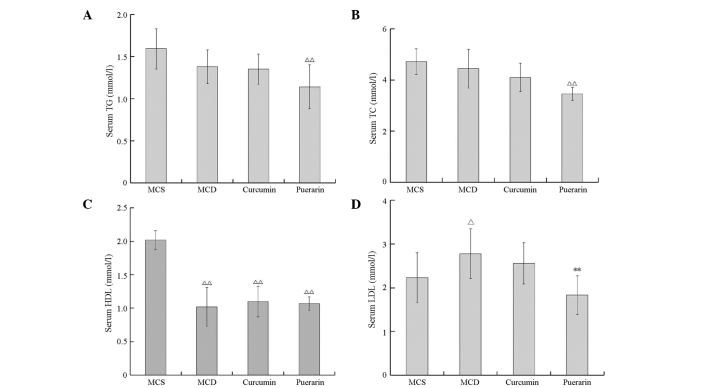
Effects of curcumin and puerarin on the concentrations of TG, TC, HDL and LDL in all experimental groups. Serum levels of (A) TG; (B) TC; (C) HDL; and (D) LDL. ^Δ^P<0.05; ^ΔΔ^P<0.01 versus the normal control (MCS) group and ^**^P<0.01 versus the MCD group (n=8 per group). MCS, methionine-choline-sufficient diet; MCD, methionine-choline-deficient diet; TG, triglyceride; TC, total cholesterol; HDL, high-density lipoprotein; LDL, low-density lipoprotein.

All animals tolerated the experimental procedures well and no deaths occurred during the 2 week study. The levels of serum TG, TC, HDL and LDL were analyzed in each group. The results showed that there were no significant differences in TG and TC levels between the normal control group and the MCD group, which indicated that the MCD diet does not induce hyperlipidemia in mice (P>0.05). Notably, puerarin treatment significantly reduced the levels of TG and TC in the serum to lower than those of the normal mice (P<0.05, versus the MCS group; Fig. 2A and B). Compared with the values in the MCS group (normal control), the HDL level decreased but the LDL level increased significantly in the MCD group (P<0.01 and P<0.05, respectively). Compared with the value in the MCD model group, the serum LDL level was reduced only in the mice treated with puerarin (P<0.01; Fig. 2C and D).

### Effects of puerarin and curcumin on the levels of TNF-α and IL-6 in mice serum

**Figure 3:**
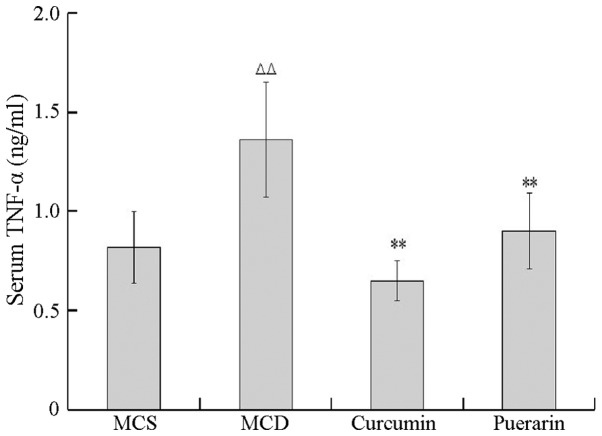
Effects of curcumin and puerarin on TNF-α levels in the serum. ^ΔΔ^P<0.01 versus the normal control group and ^**^P<0.01 versus the MCD group (n=8 per group). MCS, methionine-choline-sufficient diet; MCD, methionine-choline-deficient diet; TNF-α, tumor necrosis factor-α.

**Figure 4:**
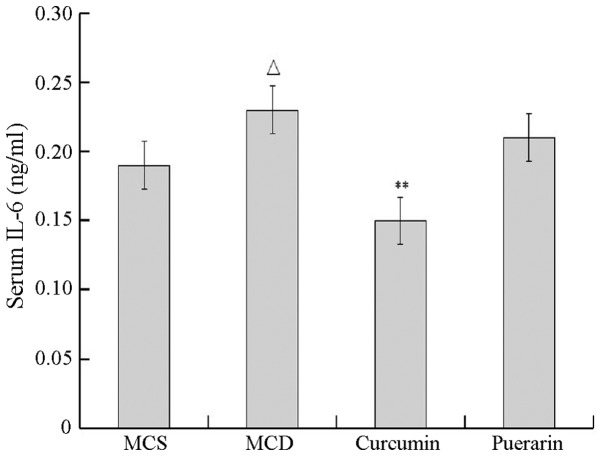
Effects of curcumin and puerarin on IL-6 levels in the serum.^Δ^P<0.05 versus the normal control group and ^**^P<0.01 versus the MCD group (n=8 per group). MCS, methionine-choline-sufficient diet; MCD, methionine-choline-deficient diet; IL-6, interleukin-6.

The inhibitory activities of puerarin and curcumin on TNF-α and IL-6 levels were tested using an ELISA method. As shown in Fig. 3, the level of TNF-α in the serum was significantly increased in the MCD group compared with that of the MCS group (P<0.01). In addition, the levels of TNF-α decreased in mice treated with curcumin and puerarin, respectively, compared with that of the MCD group (P<0.01), suggesting that curcumin and puerarin markedly inhibited TNF-α secretion. As shown in Fig. 4, the level of IL-6 also significantly increased in the MCD group compared with that of the normal control group (P<0.05). Curcumin but not puerarin inhibited IL-6 secretion compared with that of the MCD group (P<0.01).

### Histopathological evaluation

**Figure 5:**
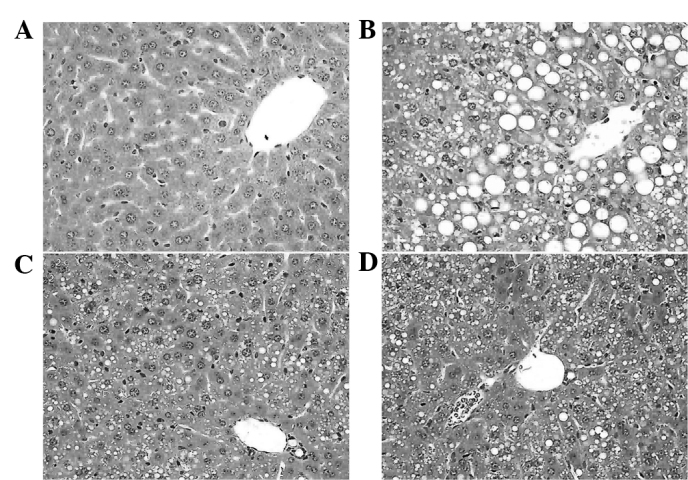
Histopathological changes of liver tissues stained with hematoxlyin and eosin in the (A) MCS; (B) MCD; (C) curcumin-treated; and (D) puerarin-treated groups (magnification, ×200). MCS, methionine-choline-sufficient diet; MCD, methionine-choline-deficient diet.

**Figure 6:**
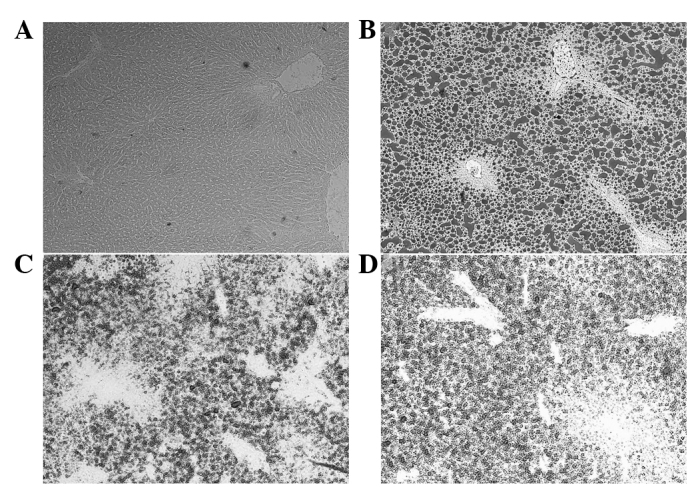
Results of Oil red O staining of frozen tissue sections (magnification, ×200). Dark staining represents lipid droplets in the hepatocytes in the (A) MCS; (B) MCD; (C) curcumin-treated; and (D) puerarin-treated groups. MCS, methionine-choline-sufficient diet; MCD, methionine-choline-deficient diet.

The histopathology of the livers was analyzed to determine whether curcumin and puerarin prevented liver destruction. As shown in Fig. 5B, typical steatosis (balloon degeneration in hepatocytes and evident infiltration with inflammatory cells in the intercellular substance) was observed in the liver tissues of the MCD group compared with that of the normal control mice (Fig. 5A). However, curcumin and puerarin treatments alleviated those changes of pathology (Fig. 5C and D), which indicated the therapeutic effects of curcumin and puerarin. Furthermore, Oil red O staining was used to detect the quantity of lipids in the hepatocytes. The results showed that the MCD diet significantly increased lipidosis in hepatocytes (Fig. 6A and B), but curcumin and puerarin treatments markedly restrained the deposition of lipid droplets in the hepatocytes (Fig. 6C and D).

### Curcumin and puerarin regulate PPARγ and NF-κB expression in MCD diet-induced mice

**Figure 7:**
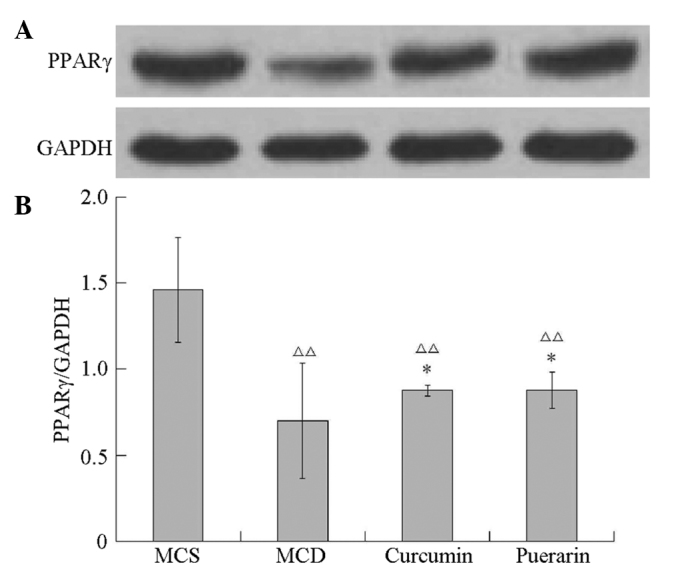
Effects of curcumin and puerarin on PPARγ expression in liver tissue homogenates. (A) Western blot analysis of the PPARγ concentration; (B) relative ratio of PPARγ. Data are expressed as the mean ± standard deviation of three independent experiments. ^ΔΔ^P<0.01 versus the normal control group and ^*^P<0.05 versus the MCD group (n=8 per group). MCS, methionine-choline-sufficient diet; MCD, methionine-choline-deficient diet; PPARγ, peroxisome proliferator-activated receptor-γ.

**Figure 8:**
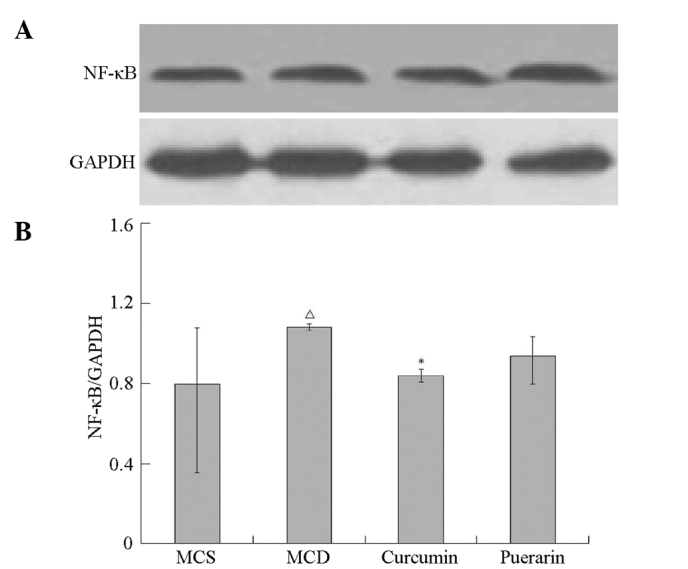
Effects of curcumin and puerarin on NF-κB expression levels in liver tissue homogenates. (A) Western blot analysis of the NF-κB concentrations; (B) relative ratio of NF-κB. Data are expressed as the means ± standard deviation of three independent experiments. ^Δ^P<0.05 versus the normal control group and ^*^P<0.05 versus the MCD group (n=8 per group). MCS, methionine-choline-sufficient diet; MCD, methionine-choline-deficient diet; NF-κB, nuclear factor-κB.

It was investigated whether curcumin and puerarin had a regulatory effect on PPARγ and NF-κB expression. As shown in Fig. 7 the PPARγ level was significantly decreased in the MCD group compared with that of the MCS group (P<0.01). Notably, curcumin and puerarin induced significant elevations of the PPARγ level compared with that in the MCD group (P<0.05). As shown in Fig. 8, NF-κB levels increased significantly in the MCD group compared with that of the MCS group (P<0.05). However, the elevation of the NF-κB level was markedly attenuated in the curcumin-treated group compared with that of the MCD group (P<0.05).

## Discussion

Nonalcoholic fatty liver disease (NAFLD) is a multi-factorial disorder resulting from a variety of genetic and environmental factors. At present, the pathogenesis of NAFLD is not fully understood and therapeutic clinical trials are ongoing (22). Although lipid accumulation in the liver is the major hallmark of NAFLD, the mechanisms resulting in steatohepatitis remain elusive (23). Researchers have suggested the ‘2-hit’ hypothesis to explain NAFLD pathogenesis (24,4). Briefly, the ‘first hit’ involves hepatic TG accumulation or steatosis. The ‘second hit’ relates to the induction of inflammatory cytokines and oxidative stress. Hepatic TG accumulation may occur as a result of increased fat synthesis, increased fat delivery, decreased fat export and/or decreased fat oxidation (25). To a certain degree, PPARγ is important for the ‘first hit’ stage (25). Inflammatory cytokines, such as TNF-α and IL-6, are involved in the ‘second hit’ stage, which mediates steatohepatitis in patients with NAFLD (26). TNF-α, a pro-inflammatory cytokine, not only promotes insulin resistance, but also mediates cholesterol and TG metabolism (27). Similarly, the involvement of IL-6 has also been identified in animal models of and patients with NAFLD, with elevated serum levels of IL-6 being correlated with increasing steatohepatitis (28). It is worth stressing that the elevated expression levels of TNF-α and IL-6 were mediated by activation of the NF-κB signaling pathway (29). NF-κB, a critical transcription factor, may promote hepatic steatosis, hepatic injury and fibrosis by upregulating serum and hepatic levels of TNF-α and IL-6 (30). An increasing number of studies have suggested that PPARγ links the NF-κB signaling pathway through being mediated by regulating adipokines via controlled by suppressing TNF-alpha and IL-6, which closely connect to NF-κB and is critical for the treatment of NAFLD (6,7,31).

In the present study, mice models of steatohepatitis induced by a MCD diet were employed to compare the efficacies of puerarin and curcumin against steatohepatitis. The MCD diet-induced animal model is an internationally-recognized model for the study of the inflammation and fibrosis associated with NAFLD (32). Methionine and choline are precursors of phosphatidylcholine (PC). PC is an essential substrate for very low density lipoproteins (VLDL). Deficiency of methionine and choline may reduce VLDL production or secretion, which limits lipid packaging and export (33,34).

As has been observed in previous studies (32,35), the MCD was noted to increased plasma TNF-α and IL-6 levels, increase hepatic tissue NF-κB expression, but decrease hepatic tissue PPARγ expression. The present study also demonstrated that there were no noticeable changes in the plasma TG levels of mice fed an MCD (32). However, the present study identified that puerarin and curcumin had promising hepatoprotective and anti-steatohepatitis activities. In addition, curcumin significantly reduced serum levels of TNF-α and IL-6, and hepatic tissue levels of NF-κB and PPARγ. The effects of puerarin differed from those of curcumin. Puerarin downregulated the serum levels of TNF-α and hepatic tissue levels of PPARγ, but demonstrated no significant effects on the levels of IL-6 and NF-κB. Moreover, compared with curcumin, puerarin indicated notable anti-hyperlipidemic effects (Fig. 2A and B). These results suggest that curcumin and puerarin affect NAFLD by different mechanisms. It is hypothesized that puerarin may be involved in the early pathological stage (first hit) through regulating the lipid metabolism mediated by PPARγ. By contrast, curcumin may impact multiple nodes, particularly the inflammatory response stage (second hit). These results suggest a novel strategy for preventing NAFLD and for the development of novel agents with anti-steatohepatitis effects.

In conclusion, curcumin and puerarin induce favorable effects on steatohepatitis through different mechanisms. Puerarin may regulate lipid metabolism in the ‘first hit’ stage through the PPARγ pathway, whereas curcumin may inhibit the inflammatory response in the ‘second hit’ stage through the PPARγ/NF-κB pathway. Further experiments focusing on the molecular mechanisms of curcumin and puerarin using different blocking agents and advanced experimental techniques are required.
